# Editorial: Next-Generation Sequencing and CRISPR-Cas Editing in Plant Virology

**DOI:** 10.3389/fmicb.2021.723278

**Published:** 2021-10-04

**Authors:** Ahmed Hadidi, Henryk Czosnek, John W. Randles

**Affiliations:** ^1^US Department of Agriculture, Agricultural Research Service, Beltsville, MD, United States; ^2^The Hebrew University of Jerusalem, Rehovot, Israel; ^3^The University of Adelaide, Waite Campus, Glen Osmond, SA, Australia

**Keywords:** next-generation sequencing, CRISPR-Cas editing, viruses, viroids, plant virus diagnosis

The Research Topic was initiated in October 2019 to demonstrate how next-generation sequencing (NGS) and clustered regularly interspaced short palindromic repeats-associated Cas protein (CRISPR-Cas) editing are being applied in plant virology. NGS combined with bioinformatics has changed both basic and applied research in many biological disciplines by its ability to deliver fast, inexpensive and accurate genome and transcriptome information (Hadidi and Barba, 2012; Barba et al., 2014). NGS has been used since 2009 in a large number of studies in plant virology, including discovery of novel viruses and viroids or their variants, sequencing the genome of known pathogens or the 21–24 nucleotide pathogen-derived small RNAs (sRNAs) generated during the infection process which frequently cover the whole virus or viroid genome. Applications include the detection and identification of pathogens, extension of their known host range, investigating pathogen-host or -vector interactions, analysis of genome diversity and evolution, mRNA targeting, symptom expression, and the study of pathogen biology (Barba et al., 2014; Hadidi et al., 2016; Hadidi, 2019; Villamor et al., 2019). In addition, metagenomics coupled with NGS of insect vectors of plant viruses allowed the discovery of virus species and variants not known to be present in the region and infecting important crops (Ng et al., 2011; Rosario et al., 2015; Fontenele et al., 2018).

CRISPR-Cas and derived systems confer adaptive immunity against bacteriophages and plasmids in many bacteria and most archaea (Dounda and Charpentier, 2014). CRISPR-Cas systems act as RNA-guided programmable nucleases to degrade DNA and/or RNA derived from foreign nucleic acids by preserving molecular memory information of prior infections (Dounda and Charpentier, 2014; O'Connell et al., 2014; Abudayyeh et al., 2016). The CRISPR-Cas-based genome editing is emerging as a powerful tool for developing plant virus-resistant crops by directly targeting the virus genome or indirectly by editing host susceptibility factors (Sanfaçon, 2015). The technique is precise in editing the target genome with and without double-stranded breaks or donor templates.

The objectives of the Research Topic were to publish high-quality research papers and review articles focusing on the following. (1) Utilization of NGS in research and diagnosis of plant viruses and viroids; (2) Direct targeting of specific nucleotide sites of plant viruses and viroids by CRISPR-based genome editing to enable the fast introduction of resistance; (3) Targeting sites of specific plant genes such as eIF4E gene or eIF (iso) 4E gene by CRISPR-Cas editing to develop plants resistant to viruses; (4) Application of the CRISPR-Cas systems for rapid and accurate diagnosis of plant viruses and viroids,. Sixteen articles have been included in this Research Topic, comprising four reviews and 12 research papers. Nine of the research papers were on plant viruses and three on viroids. Articles dealt with utilization of NGS in discovering and characterizing novel and known viruses and viroids, and applying CRISPR-Cas editing to plant translation factors for developing plants resistant to virus infection, and for rapid detection of RNA plant viruses.

## New Knowledge Gained by NGS for Novel and Known Plant Viruses and Viroids

Apple russet ring and green crinkle diseases were of undetermined etiology for many years. Using NGS, other technologies, and applying Koch's postulates, it was demonstrated that one of the sequence variants of apple chlorotic leaf spot virus causes a characteristic ring-shaped rust on fruits of infected apple trees and that a sequence variant of apple stem pitting virus causes green crinkle symptoms on an infected apple fruit. Koch's postulates were fulfilled to demonstrate the viral etiology of both apple diseases (Li et al.). It was also revealed by NGS-based analyses of mRNA and sRNA of grapevine in India that 23 known viruses and viroids occur in this host (Sidharthan et al.). The mRNA -based approach identified more pathogens than that of the sRNA. The former approach was at the same level as that of the whole transcriptome in viral identification. Genomes of 19 viruses and viroids were characterized. Identification of three recombination events and phylogenetic analyses using characterized genomes suggested possible introduction of grapevine viruses and viroids into India from several continents through infected vegetatively propagated planting material. In kiwifruit, NGS analyses revealed high molecular diversity in Actinidia virus 1 (AcV-1) populations, with the highest sequence variation among its 12 open reading frames (ORFs) occurring at ORF1a, ORF2, and ORF3 (Wen et al.). Different domain compositions were shown for the first-time in the viral ORF1a. In addition, molecular recombination events among AcV-1 variants were revealed.

In diseased *Camellia japonica*, the common camellia, five novel viruses, and two known viruses (geminivirus and blunervirus) and a large number of betaflexiviruses were discovered by NGS (Zhang et al.). NGS and phylogenetic analyses of two of the novel viruses, tentatively named camellia chlorotic ringspot viruses, suggested that they may represent a novel genus in the family *Fimoviridae*. The other three novel viruses belong to the genera *Idaeovirus, Badnavirus*, and *Marafivirus*. These findings may serve as a basis for better management of the above viruses in common camellia and possibly other hosts. NGS was also used for deciphering the virome of alfalfa plants which led to the characterization of several previously known but not fully described viruses as well as the identification of many novel viral pathogens, including alfalfa dwarf virus, alfalfa enamovirus, alfalfa leaf curl virus, alfalfa virus F., alfalfa ringspot-associated virus, and others (Bejerman et al.). Exploring the alfalfa virome by NGS has also demonstrated the impotence of viral infections in a single plant. NGS from a mixed infection of an unknown cultivar of *Veronica sp*. revealed the genome sequences of two isolates of helenium virus S (HelVS) and two distinct isolates of butterbur mosaic virus (ButMV), ButMV-A and ButMV-B. A major deletion in an essential gene of ButMV-B was identified for the first time by NGS which is maintained through complementation by ButMV-A. The HelVS host range was extended to *Veronica* sp. and it was confirmed that the virus is a distinct species in the genus *Carlavirus* (Hammond et al.). The full-length genome of a novel emaravirus was identified and characterized by NGS from diseased symptomatic sycamore maple (*Acer pseudoplatanus*)—a tree species of significance in urban and forest areas. The virus was tentatively named maple mottle-associated virus (MaMaV). Phylogenetic and sequence analyses place MaMaV in the distinct “subgroup a” clade within the *Emaravirus* genus. This is the first time an emaravirus has been described from maple and fully genetically characterized (Rumbou et al.). NGS also allowed the discovery and genome sequences of at least 70 negative-sense and ambisense RNA (NSR) plant viruses. These viruses belong to several genera in seven families: *Ophiovirus* in the family *Aspiviridae*; *Coguvirus, Rubodvirus*, and *Tenuivirus* in the family *Phenuiviridae*; *Orthotospovirus* in the family *Tospoviridae*; *Emaravirus* in the family *Fimoviridae*; *Cytorhabdovirus, Betanucleorhabdovirus, Alphanucleorhabdovirus, Dichorhavirus*, and *Varicosavirus* in the family *Rhabdoviridae*. It is predicted that the increasing use of NGS, not only for plant samples but also in arthropod vectors, will allow the identification of many novel NSR viruses which will be crucial to unraveling the evolution of many NSR virus clades (Bejerman et al.).

The 10 most abundant sequence variants of potato spindle tuber viroid (PSTVd) RG1, expressed 1–4 weeks after infecting tomato plants, were identified by NGS in the regions favoring mutations. The findings of the effect of mutations on PSTVd secondary structure and its derived small RNAs increased our knowledge of the biological role of sequence variants, PSTVd interaction with host components, stability of structures generated by mutants during the course of infection, and stabilizing viroid population dynamic as influenced by variant sequences (Adkar-Purushothama et al.). Novel circular RNAs, 357–360 nt, containing hammerhead ribozymes in both polarity strands were discovered by NGS analyses of fig tree leaves (Olmedo-Velarde et al.). Bioassays, however, are needed to demonstrate whether the RNAs are viroids or viral satellites. MicroRNAs (miRNAs) were identified by NGS in dwarfed citrus trees in response to infection by citrus dwarfing viroid (CDVd). The 60 miRNAs identified were conserved in stem and root tissues. Three conserved miRNAs (csi-miR479, csi-miR171b, and csi-miR156) were significantly downregulated in the stems of CDVd-infected trees compared to the non-infected controls. These miRNAs are known to be involved in various physiological and developmental processes some of which may be related to the characteristic dwarfed phenotype displayed by CDVd-infected sweet orange on trifoliate orange rootstock field trees. Only one miRNA (csi-miR535) was significantly downregulated in CDVd-infected roots and it was predicted to target genes controlling a wide range of cellular functions. These findings indicate that CDVd-responsive plant miRNAs play a role in regulating important citrus tree growth and developmental processes which may participate in the cellular changes leading to the observed citrus tree dwarf phenotype (Dang et al.).

## New Knowledge Gained by CRISPR-Cas Editing

### A-Developing Plants Resistant to Virus Infection

The eukaryotic translation initiation factor *eIF4E1* gene on chromosome 3 of a commercial cultivar of tomato was mutated by CRISPR/Cas9. Artificially edited alleles of *eIF4E1* gene differentially reduced susceptibility to cucumber mosaic virus and potato virus Y in tomato (Atarashi et al.). Similarly, site specific mutation of the tomato *eIF4E1* gene by CRISPR-Cas9 successfully conferred enhanced resistance to infection by pepper mottle virus (Yoon et al.). A banana streak virus (BSV) genome sequence that integrates at a single locus into the banana B genome is known as endogenous BSV (eBSV) whereas the virus genome in the replicative form in cells is known as the episomal form. CRISPR-Cas editing was applied to banana to control BSV by inactivating the eBSV integrated into the host genome (Tripathi et al.).

NGS and CRISPR-Cas editing technologies were presented in a review article that discussed in detail the development, various applications, advantages and drawbacks, as well as potential future of both technologies in plant virology (Shahid et al.).

### B-Detection of Plant RNA Viruses

**Figure 1:**
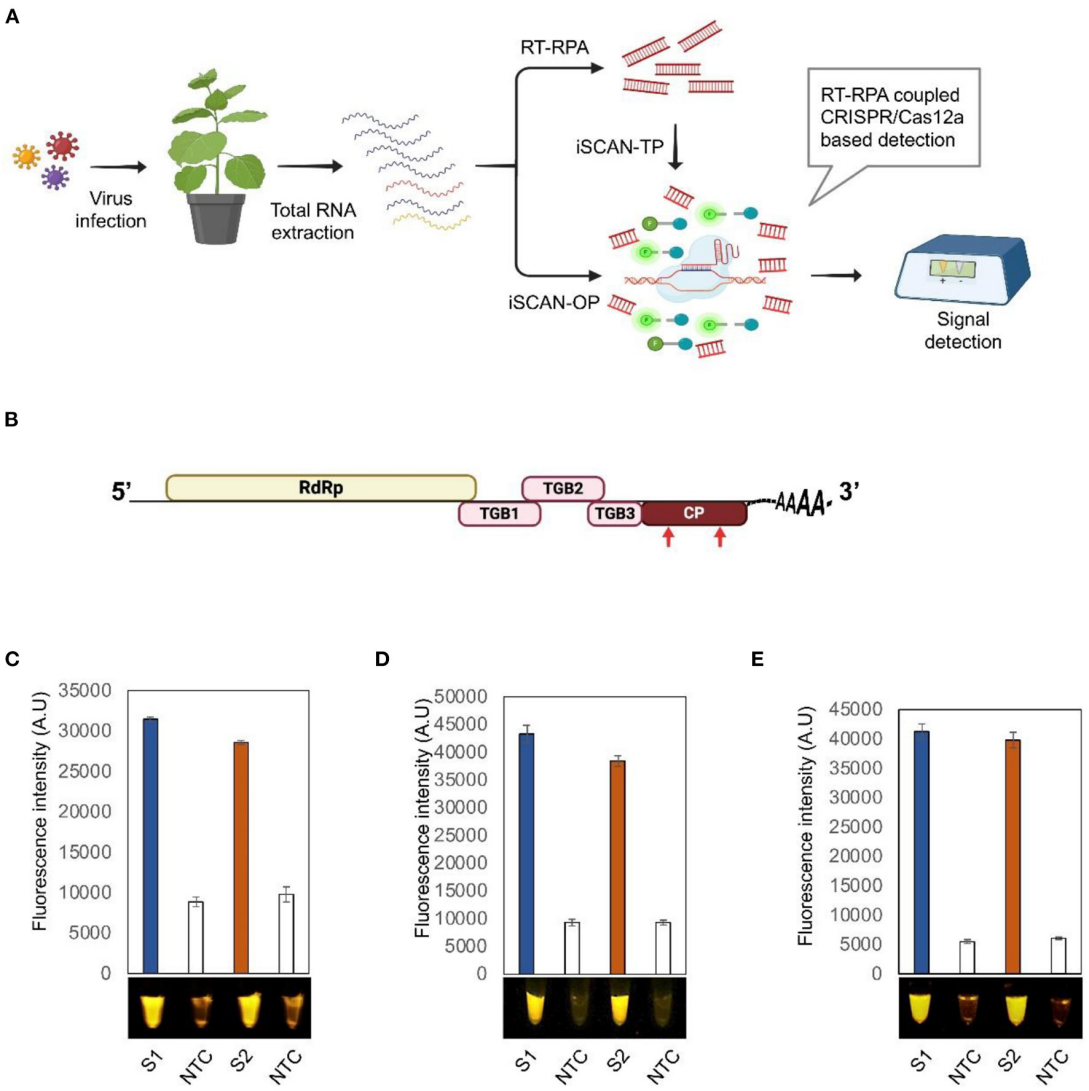
Detection of potato virus X (PVX) by RT-RPA-CRISPR/Cas12a. **(A)** Workflow of the RT-RPA coupled CRISPR/cas12a-based detection system. **(B)** Schematic diagram of PVX genome. Red arrows indicate the regions targeted by the RT-RPA CRISPR/Cas12a-based detection system. **(C–E)** End point fluorescence visualization of the iSCAN-TP (two pots) **(C)** or IScan-OP (one pot) **(D,E)** detection assay of *in vitro* transcribed RNA of PVX-CP **(C,D)** or total RNA isolated from *Nicotiana benthamiana* plants infected with PVX **(E)**. S1, RPA primer set 1; S2, RPA primer set 2; NTC, no template control. Values are shown in the graph as means ± SD (*n* = 3).

The *in vitro* Specific CRISPR-based Assay for Nucleic acids detection (iSCAN) was combined with reverse transcription-recombinase polymerase amplification (RT- RPA), to develop a one-pot detection assay termed iSCAN-one-pot (iSCAN-OP), for specific, rapid, and sensitive detection of plant RNA viruses (Aman et al.). The RT-RPA pre-amplification step converts the RNA genome of the virus into dsDNA that serves as a substrate for the Cas12a cis activity. Cas12a targeting of the dsDNA triggers its collateral activity, which in turn cleaves the ssDNA reporter molecules and releases the signal. The reaction was incubated isothermally at 42°C for 20 min. The fluorescence signal as a result of trans-cleavage activity of Cas12a was measured with a commercially available P51 Molecular Fluorescence viewer using the Tecan plate reader (Figure 1). This detection method has the potential to be used for RNA plant viruses and viroids.

## Author Contributions

AH wrote the editorial and prepared the final version of the editorial. HC and JR reviewed the editorial and made useful suggestions. All authors approved the editorial.

## Conflict of Interest

The authors declare that the research was conducted in the absence of any commercial or financial relationships that could be construed as a potential conflict of interest.

## Publisher's Note

All claims expressed in this article are solely those of the authors and do not necessarily represent those of their affiliated organizations, or those of the publisher, the editors and the reviewers. Any product that may be evaluated in this article, or claim that may be made by its manufacturer, is not guaranteed or endorsed by the publisher.
